# Maternal Factors and Nodal Autoregulation Orchestrate *Nodal* Gene Expression for Embryonic Mesendoderm Induction in the Zebrafish

**DOI:** 10.3389/fcell.2022.887987

**Published:** 2022-05-26

**Authors:** Cencan Xing, Weimin Shen, Bo Gong, Yaqi Li, Lu Yan, Anming Meng

**Affiliations:** ^1^ Laboratory of Molecular Developmental Biology, State Key Laboratory of Membrane Biology, Tsinghua-Peking Center for Life Sciences, School of Life Sciences, Tsinghua University, Beijing, China; ^2^ Daxing Research Institute, University of Science and Technology, Beijing, China; ^3^ Guangzhou National Laboratory, Guangzhou, China

**Keywords:** Nodal, Eomes, Hwa, maternal factor, mesoderm induction, zebrafish

## Abstract

Nodal proteins provide crucial signals for mesoderm and endoderm induction. In zebrafish embryos, the *nodal* genes *ndr1*/*squint* and *ndr2*/*cyclops* are implicated in mesendoderm induction. It remains elusive how *ndr1* and *ndr2* expression is regulated spatiotemporally. Here we investigated regulation of *ndr1* and *ndr2* expression using M*hwa* mutants that lack the maternal dorsal determinant Hwa with deficiency in β-catenin signaling, M*eomesa* mutants that lack maternal Eomesodermin A (Eomesa), M*eomesa*;M*hwa* double mutants, and the Nodal signaling inhibitor SB431542. We show that *ndr1* and *ndr2* expression is completely abolished in M*eomesa*;M*hwa* mutant embryos, indicating an essential role of maternal *eomesa* and *hwa*. Hwa-activated β-catenin signaling plays a major role in activation of *ndr1* expression in the dorsal blastodermal margin, while *eomesa* is mostly responsible for *ndr1* expression in the lateroventral margin and Nodal signaling contributes to ventral expansion of the *ndr1* expression domain. However, *ndr2* expression mainly depends on maternal *eomesa* with minor or negligible contribution of maternal *hwa* and Nodal autoregulation. These mechanisms may help understand regulation of Nodal expression in other species.

## Introduction

The *Nodal* gene was first identified in mouse and its encoded protein belongs to a member of transforming growth factor β (TGFβ) family (Zhou et al., 1993). Disruption of the mouse *Nodal* gene results in failure of primitive streak formation and mesoderm induction during embryonic development (Zhou et al., 1993; Conlon et al., 1994). There are three *nodal* genes in the zebrafish genome, *ndr1/squint* (*sqt*) (Erter et al., 1998; Rebagliati et al., 1998), *ndr2/cyclops* (*cyc*) (Rebagliati et al., 1998), and *ndr3/southpaw* (*spaw*) (Long et al., 2003). Simultaneous deficiency of zebrafish zygotic *ndr1* and *ndr2*, which is caused by gene mutations, leads to loss of most, if not all, endodermal and mesodermal tissues (Feldman et al., 1998), indicating that these two Nodal proteins produced zygotically are mesendoderm inducers during zebrafish embryogenesis. Interestingly, *ndr1* is also maternally expressed with maternal transcripts localized in the presumptive dorsal blastomeres during cleavage period (Gore and Sampath, 2002; Gore et al., 2005); it is believed that maternal *ndr1* transcripts act as scaffold noncoding RNAs to spatially regulate β-catenin signaling (Lim et al., 2012). Maternal *ndr1* has been shown to cooperate with extraembryonic (yolk syncytial layer) *ndr1* and extraembryonic *ndr2* to specify endoderm and anterior mesoderm fates (Hong et al., 2011), but it remains elusive if this function of maternal *ndr1* is executed through classical Nodal signaling. The zebrafish *ndr3* gene is not expressed until the completion of gastrulation, and it is required for left-right asymmetrical development after the completion of gastrulation (Long et al., 2003; Hashimoto et al., 2004; Zhang et al., 2012; Zhang et al., 2016). The importance of Nodal signaling in mesendoderm induction has also been revealed in frog embryos (Jones et al., 1995; Joseph and Melton, 1997; Osada and Wright, 1999; Agius et al., 2000; Takahashi et al., 2000; Luxardi et al., 2010). It is now widely accepted that zygotically expressed Nodal proteins are essential for mesendoderm induction and patterning in vertebrate embryos (Schier and Shen, 2000; De Robertis and Kuroda, 2004; Tian and Meng, 2006; Zinski et al., 2018).

In frog and fish embryos, mesendoderm induction occurs during middle to late blastulation. As essential mesendoderm inducers, the expression of zygotic *nodal* genes is activated by maternal factors soon after midblastula transition (MBT), which happens in zebrafish embryos around 3 h postfertilization (hpf) (1 k-cell stage) (Kimmel et al., 1995). In frog blastulas, the maternal T-box transcription factor VegT activates the expression of *Xenopus* Nodal-related (*Xnr*) genes in the vegetal blastomeres and maternally regulated nuclear β-catenin in dorsal blastomeres acts in synergy with VegT to enhance *Xnr* genes expression so that a Nodal gradient is formed along the dorsal-ventral axis to induce and pattern the mesendoderm (Zhang et al., 1998; Kofron et al., 1999; Agius et al., 2000; Takahashi et al., 2000; Rex et al., 2002; Xanthos et al., 2002). In the zebrafish, *ndr1* and *ndr2* genes are initially activated in the dorsal blastodermal margin at about 3.3 h hpf and 3.7 hpf respectively, and their expression domains then extend throughout the blastodermal margin to induce the mesendodermal fate (Feldman et al., 1998; Rebagliati et al., 1998). β-catenin signaling plays a role in activating *ndr1* and *ndr2* expression in the dorsal blastodermal margin (Kelly et al., 2000; Dougan et al., 2003; Bellipanni et al., 2006). It has been recently disclosed that β-catenin signaling is activated by maternal *huluwa* (*hwa*), which encodes a transmembrane protein, in zebrafish and *Xenopus* blastulas (Yan et al., 2018). Maternal *hwa* transcripts in both species are located in the vegetal pole of the mature oocyte. Upon fertilization in *Xenopus*, maternal *hwa* transcripts shift to one side with cortical rotation and are apparently enriched in the dorsal blastomere at 2-cell stage; after fertilization in zebrafish, *hwa* transcripts in the vegetal pole transport to the cytoplasm in the animal pole and become ubiquitously distributed in blastulas, but Hwa protein is located in a few blastomeres in the prospective dorsal side at 2.75 hpf (Yan et al., 2018). Zebrafish M*hwa* mutants are severely ventralized (Yan et al., 2018), which are similar to the most severe phenotype (Class I) in *β-catenin2* deficient *ichabod* mutants (Kelly et al., 2000). The zebrafish T-box transcription factor Eomesodermin a (Eomesa) is maternally expressed with a vegetal-to-animal gradient distribution of transcripts during cleavage period and around MBT stages (Bruce et al., 2003). We previously demonstrate that zygotic expression of zebrafish *ndr1* and *ndr2* also requires Eomesa, in particular in ventral and lateral blastodermal margins, which is then assumed to be a zebrafish functional counterpart of frog VegT (Xu P. et al., 2014). It remains genetically unverified whether VegT/Eomesa and Hwa/β-catenin signaling are essential maternal factors for zygotic *nodal* genes expression in vertebrate embryos, and if they are, it needs to be investigated whether they differentially contribute to initiation, range and level of zygotic *nodal* genes expression.

Nodal proteins bind to specific receptors on the cytoplasm membrane, which recruit and phosphorylate the intracellular effectors Smad2 and Smad3 (Tian and Meng, 2006; Shen, 2007). The activated Smad2/3 (p-Smad2/3) bind to Smad4 and the formed complexes translocate into the nucleus to activate, with help of FoxH1 or/and other transcription factors, target genes expression. Studies in model animals have disclosed that *nodal* genes themselves contain Nodal-responsive elements (Adachi et al., 1999; Norris and Robertson, 1999; Osada et al., 2000; Fan et al., 2007; Liu et al., 2011), implying that Nodal signaling reinforces itself *via* positive feedback regulation. On the other hand, as diffusible proteins (Jones et al., 1996; Chen and Schier, 2002; Schier, 2009; Muller et al., 2012; Muller et al., 2013), Nodal proteins produced in one area are able to transduce the signal to neighboring areas that previously lack *nodal* transcripts to initiate *nodal* gene expression for self-propagation or relay (Meno et al., 1999; Hyde and Old, 2000; Brennan et al., 2001; Chen and Schier, 2001; Dougan et al., 2003; Williams et al., 2004; Muller et al., 2012; Xu P.-F. et al., 2014). However, it is unclear how much autoregulation of Nodal signaling adds to Nodal activity during vertebrate mesendoderm induction.

In this study, we systematically investigated spatiotemporal regulation of zygotic *ndr1* and *ndr2* expression during mesendoderm induction. We show that maternal Eomesa, maternal Hwa-activated β-catenin signaling, and Nodal positive self-regulation, are required, but to different degrees, for correct spatiotemporal expression of *ndr1* and *ndr2*.

## Materials and Methods

### Zebrafish Strains and Embryo Incubation

**FIGURE 1:**
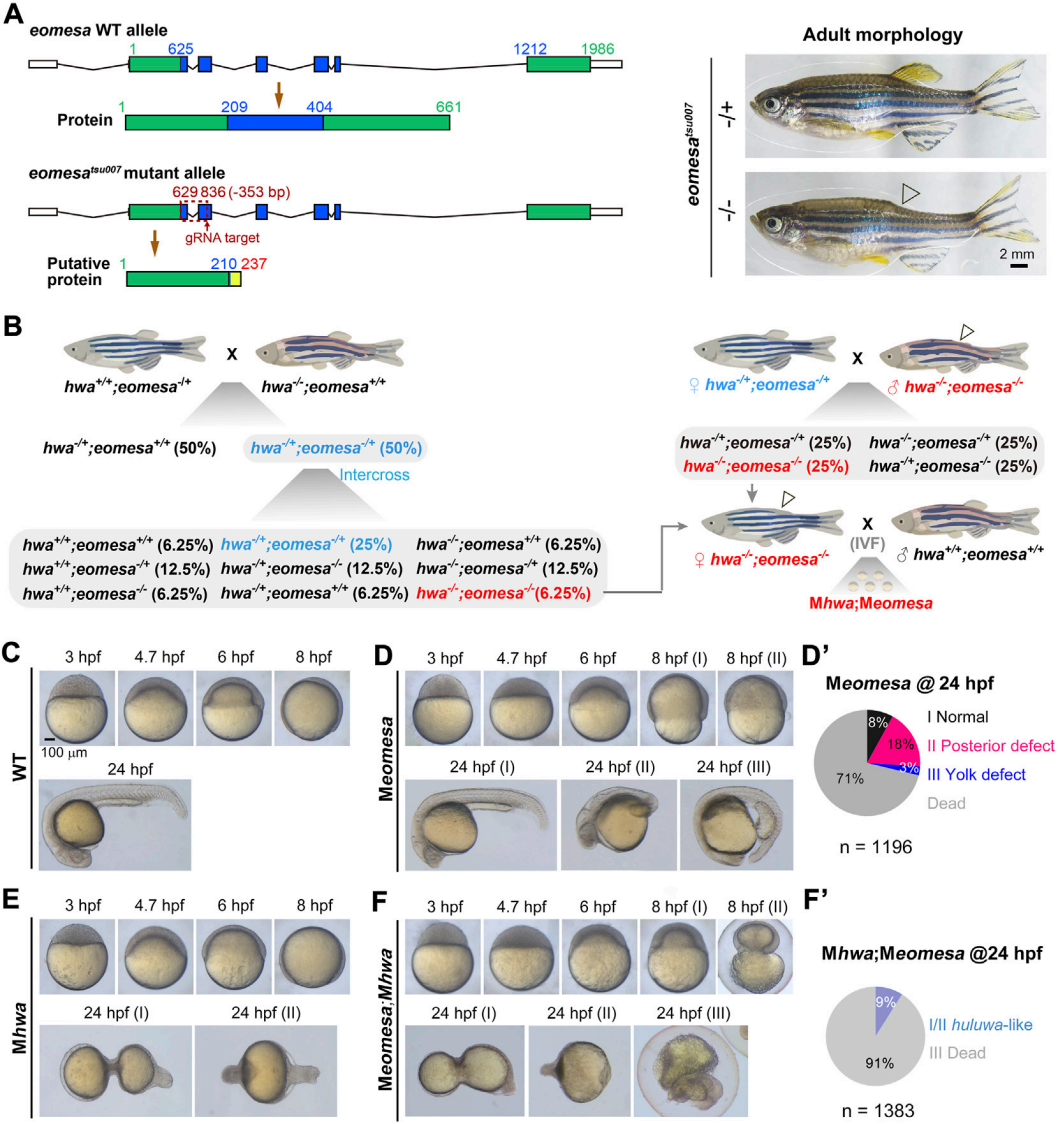
Phenotypes of different mutants at various stages. **(A)** Generation of *eomesa* ^*tsu007*^ mutant allele. Left: genomic structures and putative coding products of *eomesa* WT allele and tsu007 allele. The exons were colored and positions of nucleotides and amino acids were indicated. The mutant allele carries a 353-bp deletion. Right: morphology of *eomesa* ^*+/tsu007*^ (heterozygote) and *eomesa* ^*tsu007/tsu007*^ (zygotic mutant) adults. Note that the posterior dorsal fin (indicated by an hollow arrowhead) is absent in mutant adult. **(B)** Scheme of generation of M*hwa;*M*eomesa* double mutants. Zygotic genotypes were indicated. Z*hwa* ^*−/−*^ *;*Z*eomesa* ^*−/−*^ homozygous female adults could not ovulate naturally, and eggs squeezed from these fish were used for *in vitro* fertilization (IVF) using sperms squeezed from wildtype males (Z*hwa* ^*+/+*^ *;*Z*eomesa* ^*+/+*^). **(C)** Wildtype (WT) embryos at indicated stages. **(D,D’)** M*eomesa* mutants. Mutant embryos exhibited variable phenotypes at 8 hpf and 24 hpf, and the ratios of 24-hpf mutants categorized into different classes (I-III), which were derived from several homozygous females, were shown in **(D’)**. **(E)** M*hwa* mutants at indicated stages. **(F,F’)** M*eomesa;*M*hwa* double mutants. The ratios of embryos with different phenotypes at 24 hpf were shown in **(F’)**. Embryos were laterally positioned when the dorsal or tail was recognizable. The scale bar in **(C)** was also applied to **(D–F).**

The zebrafish Tuebingen strain was used as wildtype fish and for generating mutants. The *eomesa* ^*tsu007*^ mutant line that carries a 353-bp deletion was generated by the CRISPR-Cas9 system with a gRNA (5′-ggc​gga​aag​tgg​gtg​acc​tgc​gg-3′) targeting the second exon of *eomesa* (Figure 1A). For genotyping the *eomesa* ^*tsu007*^ mutant allele, the upper primer (5′- CTC​AGC​TCG​ATG​CCC​ATT​C-3′) and the lower primer (5′- ATA​CAG​TCT​TTG​TCG​GAG​ATG-3′) were used for PCR. Like the previously reported *eomesa* ^*fh105*^ mutant line (Du et al., 2012), zygotic *eomesa* ^*tsu007*^ (Z*eomesa* ^*tsu007*^) homozygous embryos were able to grow up to adulthood with loss of the dorsal fin (Figure 1A). The *hwa* ^*tsu01sm*^ mutant line was used and its genotyping was described before (Yan et al., 2018). The *eomesa* ^*tsu007/+*^ *;hwa* ^*tsu01sm/+*^ double heterozygotes were obtained by crossing *eomesa* ^*tsu007/+*^ female to *hwa* ^*tsu01sm/+*^ male. Then, Z*eomesa* ^*tsu007/tsu007*^ *;*Z*hwa* ^*tsu01sm/tsu01sm*^ double homozygous fish (i.e., Z*eomesa*;Z*hwa* double homozygotes) were obtained by intercrossing the double heterozygotes (Figure 1B). Like Z*eomesa* mutant female that were unable to naturally ovulate (Du et al., 2012; Xu P. et al., 2014), Z*eomesa*;Z*hwa* double homozygous female were unable to naturally ovulate and *in vitro* fertilization using their squeezed eggs and wildtype male-derived sperms were performed to obtain maternal double mutant (M*eomesa*;M*hwa*) embryos (Figure 1B).

Embryos were maintained in Holtfreter’s water at 28.5°C. Developmental stages of WT embryos were determined as described before (Kimmel et al., 1995) while those of mutants were indirectly judged by developmental time matching to WT embryos in the same conditions. Embryo treatment with SB431542 (SB) were performed as described before (Sun et al., 2006). Briefly, One-cell stage embryos of different mutant or WT lines were incubated in Holtfreter’s water with addition of freshly made SB to a final concentration of 50 μM in a dish and then harvested for observation or assays at desired stages. All experiments were approved by Tsinghua University Animal Care and Use Committee.

### Constructs and Microinjection

The plasmids *pCS2-eomesa-Myc* (Bruce et al., 2003) and *pCS2-hwa-HA* (Yan et al., 2018) were used to *in vitro* synthesize capped mRNAs using mMESSAGE mMACHINE Kit (Ambion). To knock down zebrafish *β-catenin2*, β-cat2-MO and its control morpholino (cMO) were used as described before (Zhang et al., 2012). mRNA or MO was injected into embryos at the one-cell stage.

### Quantitative RT-PCR

Embryos or eggs (15 per sample) were harvested at desired stages, and used to extract total RNA by RNeasy Mini Kit (Qiagen) as previously described (Jia et al., 2009). cDNA was synthesized using the M-MLV reverse transcriptase (Promega) and qRT-PCR was performed with TransStart Top Green qPCR SuperMix (TransGen Biotech) as described (Sun et al., 2018). Expression levels were normalized to the reference gene *eif4g2a* unless otherwise stated. The Student’s *t*-test (two-tailed, unequal variance) was used to determine *p*-values. Primers and sequences for qRT-PCR analysis were as follows: ndr1-F (5′-TTG​GAT​ATG​CTC​CTT​GAC​CC-3′), ndr1-R (5′-ACA​GAT​AAG​GCA​AAC​ACG​CAA​A-3′); ndr2-F (5′-GAA​ATA​TCA​TCA​CCC​CAG​TCG​T-3′), ndr2-R (5′-CTC​CAC​CTG​CAT​GTC​CTC​GT-3′); tbxta-F (5′-TTG​GAA​CAA​CTT​GAG​GGT​GA-3′), tbxta-R (5′-CGG​TCA​CTT​TTC​AAA​GCG​TAT-3′); sox32-F (5′-TCT​GCC​ACG​GTC​TGC​TTA​C-3′), sox32-R (5′-CAG​AGA​AGG​TCC​ACC​CAA​AC-3′); gata2a-F (5′-CTC​CTC​AGC​GGA​TCC​GCT​TCC​AGC-3′), gata2a-R (5′-GGT​CGT​GGT​TGT​CTG​GCA​GTT​CGC-3′); gsc-F (5′-GAG​ACG​ACA​CCG​AAC​CAT​TT-3′), gsc-R (5′-CCT​CTG​ACG​ACG​ACC​TTT​TC-3′).

### Statistics

The graphs and *t*-test were finished with GraphPad Prism 7. Error bars represent mean ± SD. *p* values are two-sided. Significance levels were indicated by nonsignificant (ns); *, *p* < 0.05; **, *p* < 0.01; ***, *p* < 0.001.

## Results

### Loss of Mesendodermal Fates and *Nodal* Genes Expression in M*eomesa*;M*hwa* Double Mutants

The *eomesa* ^*tsu007*^ mutant allele harbors a 353-bp deletion between the first and the second exon, resulting in a premature stop codon upstream of the T-box coding region (Figure 1A). Maternal *eomesa* mutants (M*eomesa* ^*tsu007*^) showed delayed epibolic process; the majority of M*eomesa* ^*tsu007*^ mutants died before 24 hpf and survivors at 24 hpf had a normal head with thin posterior trunk (posterior defect) or had thin anterior trunk with a bulged yolk extension (yolk defect) (Figures 1C-D’). These defects are similar to those observed in M*eomesa* ^*fh105*^ mutants (Du et al., 2012).

Using *eomesa* ^*tsu007*^ and *hwa* ^*tsu01sm*^ lines (Yan et al., 2018), we managed to obtain *eomesa;hwa* double homozygotes (Z*eomesa;*Z*hwa*) female fish, which were fertile and used to produce M*eomesa;*M*hwa* embryos by *in vitro* fertilization using sperms squeezed from WT males. Generally, the M*eomesa*;M*hwa* double mutants exhibited more severe phenotype than either of single mutants (Figures 1D–F). An average of 91% maternal double mutant embryos were arrested and deformed during gastrulation and the remaining embryos at 24 hpf had a degenerating tail-like structure with missing of other tissues such as head and anterior trunk (Figures 1F, F’), indicating cooperative roles of maternal *eomesa* and *hwa* in embryonic survival.

**FIGURE 2:**
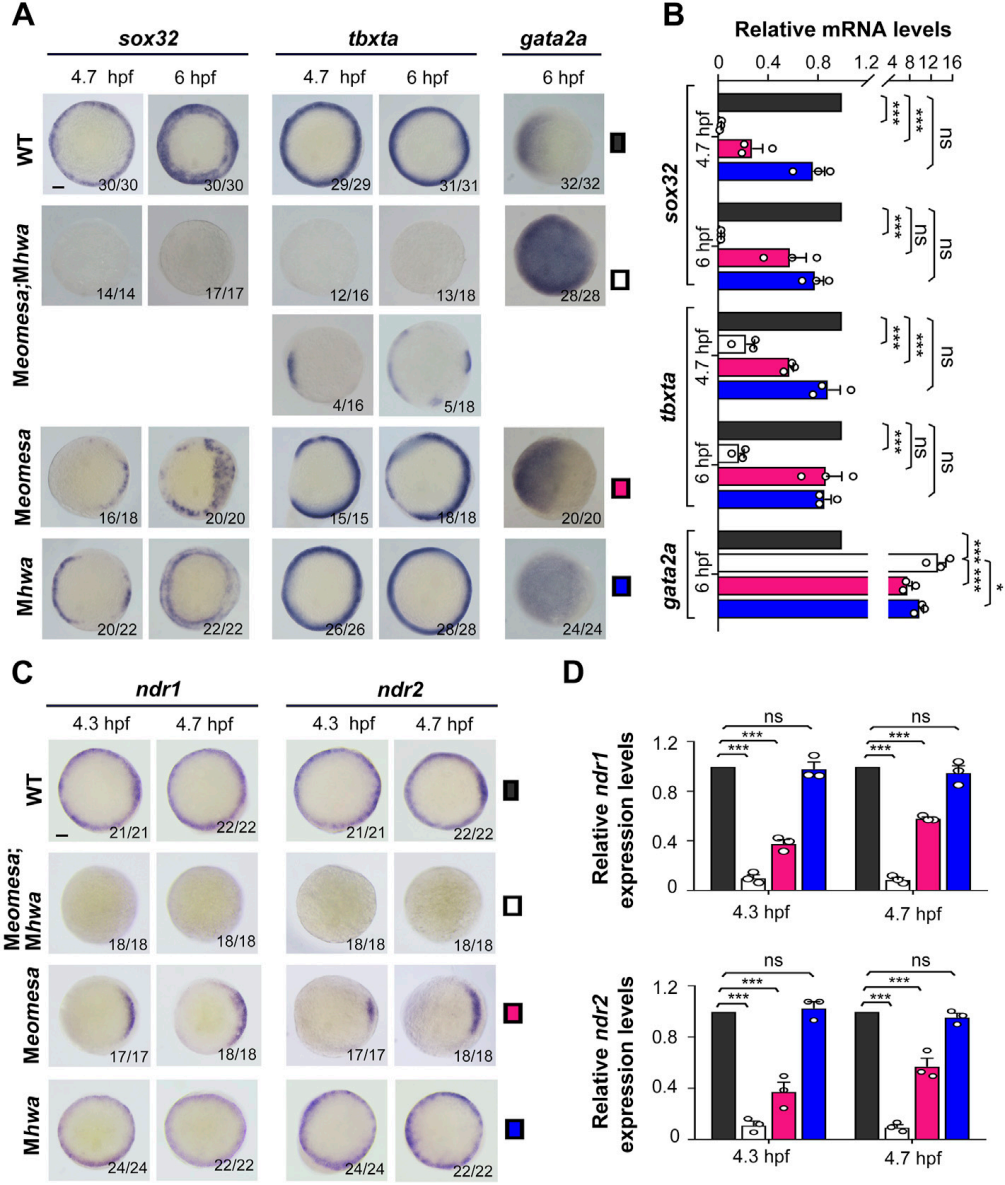
Expression patterns of mesendodermal markers and *nodal* genes in WT and mutant embryos. The expression of the endodermal marker *sox32*, the mesodermal marker *tbxta* and the epidermal marker *gata2a* as well as *ndr1* and *ndr2* was examined by WISH **(A,C)** or qRT-PCR **(B,D)** at indicated stages. Embryos in **(A,C)** were shown in animal-pole view with dorsal to the right if the dorsal was recognizable. The ratio of embryos with the representative pattern was indicated at the right bottom. Note that the majority of M*eomesa*;M*hwa* embryos completely lacked *tbxta* expression while the other had some *tbxta* expression. Scale bars: 100 μm. For RT-PCR analysis, 15 embryos were pooled for each assay, and the expression level was normalized to that of *eif4g2a* in WT embryos at the same stage*.* Error bars indicated S.D. based on three biological replicates (indicated by small circles). Color keys for embryo types were shown in **(A,C)**. Statistically significant levels: ns, nonsignificant; **, *p* < 0.01; ***, *p* < 0.001.

Then, we examined expression patterns of the endodermal marker *sox32*, the mesodermal marker *tbxta* (previously named *ntla*) and the epidermal marker *gata2a* in the single and double mutants at 4.7 hpf (30% epiboly stage) and 6 hpf (shield stage) by whole mount *in situ* hybridization (WISH) (Figure 2A). Compared to WT embryos, *sox32* expression in either of single mutants was weaker with some missing domains in the blastodermal margin, whereas it was completely abolished in M*eomesa;*M*hwa* double mutants. M*eomesa* mutants showed missing of *tbxta* expression in some portions of the margin, which is consistent with the pattern observed in M*eomesa* ^*fh105*^ embryos (Xu P. et al., 2014), and M*hwa* mutants appeared to express *tbxta* in the whole margin; in contrast, 73% of M*eomesa;*M*hwa* double mutants lacked *tbxta* expression and the remaining proportion had a few small *tbxta*-expressing patches that might be caused by evoked genetic compensation through unknown mechanisms. The expression domain of *gata2a* was dorsally expanded in either of single mutants but further expanded throughout the blastoderm in the double mutants. qRT-PCR analysis using specific primers revealed a drastic decrease of *sox32* and *tbxta* expression levels with a concomitant increase of *gata2a* levels in the double mutants (Figure 2B). These results indicate that maternal *eomesa* and *hwa* are two essential genes for mesendoderm induction and the whole blastoderm with their simultaneous loss may acquire the *epidermis* fate.

Given that Nodal signaling is critical for mesendoderm induction, we wondered how *ndr1* and *ndr2* expression were altered in M*eomesa;*M*hwa* double mutants. WISH results showed that either *ndr1* or *ndr2* expression was undetectable in the double mutants at 4.3 hpf and 4.7 hpf while detected in either of the single mutants (Figure 2C). qRT-PCR analyses using embryo pools disclosed that, compared to those in WT embryos, *ndr1* and *ndr2* levels were decreased significantly in M*eomesa* mutant embryos and further dropped in M*eomesa*;M*hwa* double mutants whereas their expression levels were not changed significantly in M*hwa* embryos (Figure 2D). These results indicate that *ndr1* and *ndr2* expression is initiated in the absence of either maternal *eomesa* or *hwa* but fail to initiate in the absence of both maternal factors.

### *eomesa* or *hwa* Overexpression Distinctly Activates *ndr1* and *ndr2* Expression in M*eomesa*;M*hwa* Double Mutants

**FIGURE 3:**
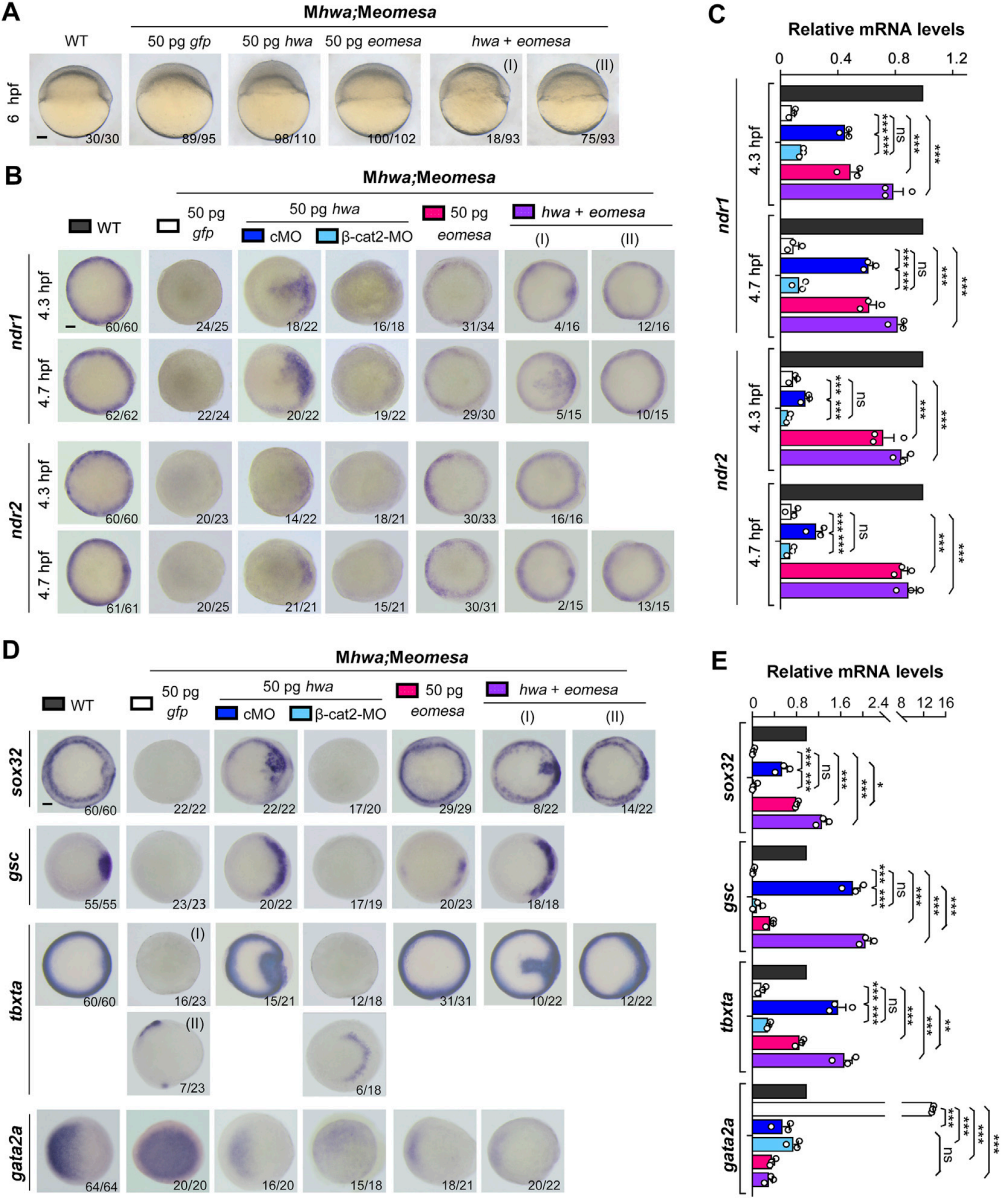
Induction of *nodal* genes and mesendodermal markers in M*eomesa;*M*hwa* mutants by ectopic *hwa* or/and *eomesa.* One-cell stage mutant embryos were injected with corresponding mRNA or/and MO and observed for morphology at 6 hpf **(A)** or harvested at indicated stages for detection of selected genes by WISH **(B,D)** or by RT-PCR analysis **(C,E)**. Embryos were positioned laterally **(A)** or in animal-pole view **(B,D)** with dorsal to the right if the dorsal side was perceptible. The ratio of embryos with the representative pattern was indicated in the right bottom. Scale bars: 100 μm. Injection doses of mRNA or MO: *hwa* and *myc-eomesa*, 50 pg/embryo; cMO (as control MO) and β-cat2-MO, 20 ng/embryo. qRT-PCR analysis was performed using 15 embryos per sample, and the expression level was normalized to that of *eif4g2a* in WT embryos at the same stage. Error bars indicated S.D. based on three biological replicates (indicated by small circles). Color keys for embryo types and treatments were shown in **(A,B,D)**. Statistically significant level: ns, nonsignificant; *, *p* < 0.05; **, *p* < 0.01; ***, *p* < 0.001.

Next, we tested the capability of exogenous *eomesa* and *hwa* to induce *ndr1* and *ndr2* in the absence of both endogenous Eomesa and Hwa. We injected *myc-eomesa*, *hwa* or both mRNAs into M*eomesa*;M*hwa* double mutant embryos at the one-cell stage. Morphological observation at 6 hpf indicated that *eomesa* but not *hwa* overexpression could largely rescue the M*eomesa*;M*hwa* phenotype of slow epiboly, and co-overexpression of *eomesa* and *hwa* could restore the embryonic shield (Figure 3A). The injected embryos were then examined for *ndr1* and *ndr2* expression at 4.3 hpf and 4.7 hpf by WISH. Results disclosed that *myc-eomesa* overexpression induced *ndr1* and *ndr2* expression in the whole blastodermal margin, *hwa* overexpression activated their expression only in one side of the blastoderm (presumably dorsal side), and co-overexpression induced their expression at higher levels (Figure 3B). The induction of *ndr1* and *ndr2* by *hwa* mRNA was abolished when *β-cat2* was knocked down with an antisense morpholino, which corroborates that *hwa* mainly exerts its effect through activation of β-catenin signaling (Yan et al., 2018). Besides, *hwa* showed a stronger induction activity for *ndr1* than for *ndr2* while *eomesa* had a stronger induction activity for *ndr2* than for *ndr1*. These observations were confirmed by qRT-PCR data (Figure 3C). These results imply that either Eomesa or Hwa is sufficient to activate *nodal* genes expression, however, the former may be a more general activator while the latter may act as a regional activator.

We then investigated mesendoderm induction capacity of *eomesa* and *hwa* in maternal double mutants. We found that overexpression of *eomesa* or *hwa* alone or together in M*eomesa;*M*hwa* embryos could induce expression of *sox32*, *gsc* and *tbxta* but reduce *gata2* expression as examined by WISH and qRT-PCR (Figures 3D,E). Notably, overexpression effect of *hwa* was eliminated (on *sox32*, *gsc* and *tbxta*) or reduced (on *tbxta* and *gata2a*) when *β-catenin2* was knocked down at the same time, which confirmed dependence of *hwa* function on β-catenin2 (Yan et al., 2018). Besides, compared to *hwa*, ectopic *eomesa* exhibited a stronger inductive effect on *sox32* and *tbxta* but weaker effect on the dorsal mesodermal marker *gsc*, supporting the idea that *eomesa* plays a more general role in mesendoderm induction.

**FIGURE 4:**
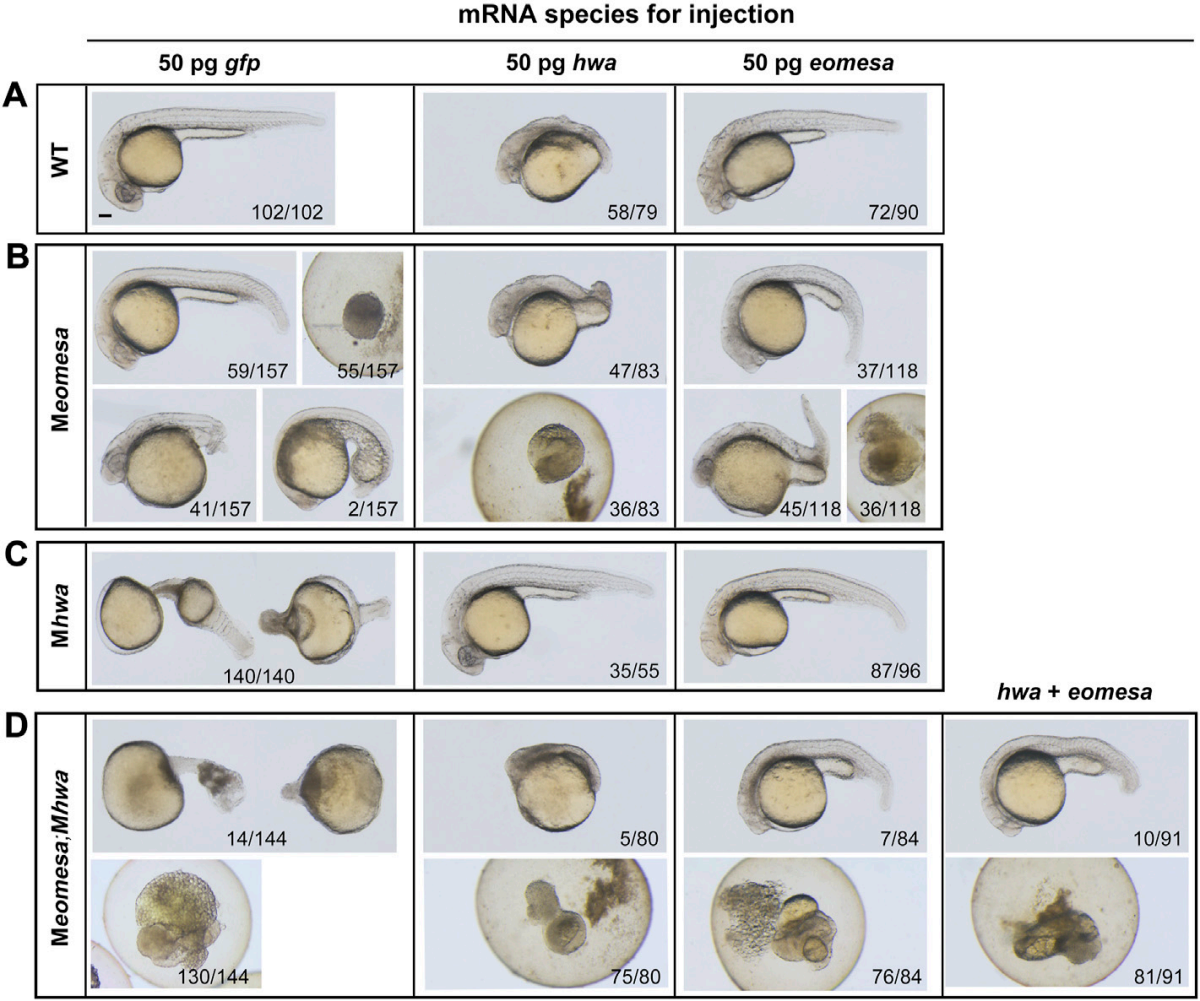
Overexpression effect of *eomesa* and *hwa* in WT and different types of mutant embryos on morphological changes at 24 hpf. **(A–D)** Morphology of WT or mutants at 24 hpf under different mRNAs injection. One-cell stage WT or mutant embryos were injected with corresponding mRNA and observed for morphology at 24 hpf. Embryos were positioned laterally with the head to the left. The ratio of embryos with the representative pattern was indicated at bottom. Injection doses: *hwa* and *myc-eomesa*, 50 pg/embryo. Scale bars: 100 μm.

We extended our observation to morphological changes in WT, M*hwa*, M*eomesa*, or M*eomesa*;M*hwa* embryos at 24 hpf after overexpression of *eomesa, hwa* or together. Generally, *hwa* overexpression in WT embryos led to strong embryonic dorsalization with missing posterior structures as reported before (Yan et al., 2018), whereas *eomesa* overexpression caused relatively weak dorsalization with thinner posterior structures (Figure 4A). Notably, 90.6% (n = 96) of M*hwa* mutants, which lack the head and anterior trunk structures (Yan et al., 2018), were able to form the head and the whole trunk following *eomesa* overexpression (Figure 4C), while *hwa* overexpression in M*eomesa* mutants still caused strong dorsalized phenotype (Figure 4B). As described above, most of M*eomesa*;M*hwa* double mutants died before 24 hpf and the survivors all had a degenerating tail-like structure without a head; however, overexpression of *eomesa* or *hwa* alone or together appeared unable to evidently reduce the mortality (Figure 4D). Nevertheless, *eomesa* overexpression alone or co-overexpression with *hwa* allowed 8–11% of embryos to form the head and the trunk, whereas *hwa* overexpression alone allowed only 6.3% of embryos to form an abnormal head and anterior trunk with missing of posterior trunk structures (Figure 4D). Although the above overexpression effects should be investigated further by titrating dosages of ectopic mRNA species, our observations support an idea that the role of *eomesa* in development of ventrolateral mesendoderm-derived tissues may not be replaced by *hwa*.

### Inhibition of Nodal Signaling Impairs Mesendoderm Induction in Mutant Embryos

**FIGURE 5:**
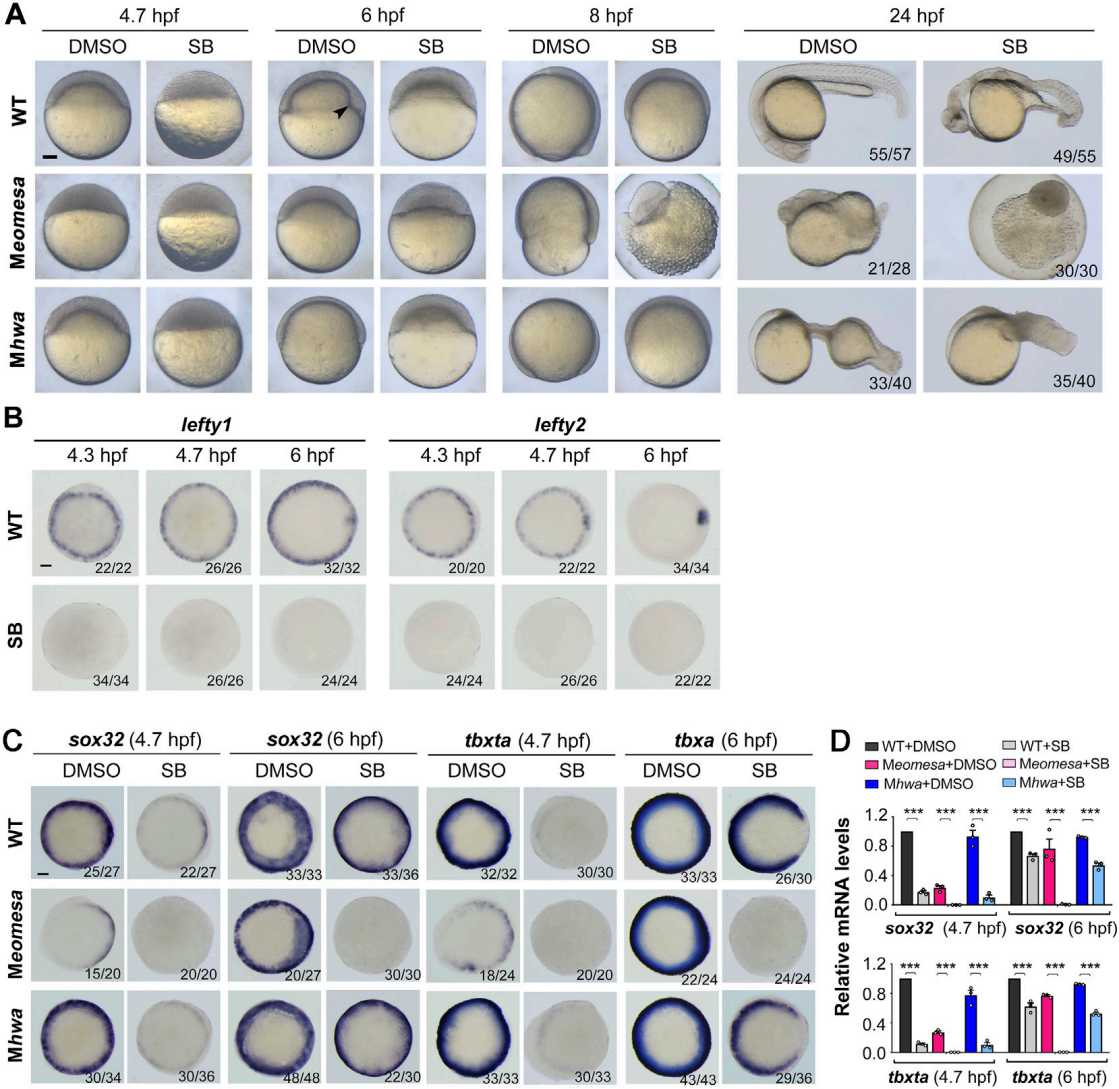
Responses of M*eomesa* and M*hwa* mutants to Nodal signaling inhibition. One-cell stage embryos (10 min postfertilization) were incubated in Holfreter’s water with 1% DMSO (control) or 50 μM SB431542 (SB, the Nodal signaling inhibitor), and harvested at 24 hpf for morphological observation **(A)** or for detection of marker gene expression by WISH **(B,C)** or qRT-PCR analysis **(D)** at indicated stages. Note that inhibition of Nodal signaling aggravated mesendodermal defects in both M*eomesa* and M*hwa* mutants **(A)**. Embryos were positioned laterally **(A)** or in animal-pole view with dorsal to the right **(B,C)** if the dorsal or tail was perceptible. The embryonic shield in WT embryo at the shield stage was indicated by an arrowhead. Scale bars, 100 μm. The ratio of embryos with the representative pattern was indicated in the right bottom **(B,C)**. qRT-PCR analysis was performed using 15 embryos per sample, and the expression level was normalized to that of *eif4g2a* in WT embryos at the same stage. Error bars indicated S.D. based on three biological replicates (indicated by small circles). Statistically significant level: ***, *p* < 0.001.

Previous studies have demonstrated that the TGFβ signaling inhibitor SB431542 (SB) or SB505124 can efficiently block Nodal signaling, resulting in loss of mesendodermal tissues in zebrafish embryos (Sun et al., 2006; Hagos and Dougan, 2007). We set out to look into effect of Nodal signaling inhibition on mesendodermal induction in M*eomesa*, M*hwa* or M*eomesa*;M*hwa* mutant embryos. One-cell stage embryos of different mutant or WT lines were incubated in the presence of 50 μM SB until harvested for observation or assays. As shown before (Sun et al., 2006), SB treatment caused loss of the embryonic shield at the shield stage (6 hpf) and most mesendodermal tissues in WT embryos at 24 hpf (Figure 5A). SB-treated M*hwa* or M*eomesa* mutant embryos at 24 hpf also showed more severe defects compared to the untreated control mutants (Figure 5A). The complete loss of the Nodal target genes *lefty1* and *lefty2* in SB-treated WT embryos confirmed the effectiveness of SB treatment (Figure 5B). Then, we examined expression of *sox32* and *tbxta* at 4.7 hpf and 6 hpf by WISH and qRT-PCR analysis (Figures 5C,D). WISH results showed that *sox32* and *tbxta* expression became very weak at 4.7 hpf and largely recovered at 6 hpf in SB-treated WT and M*hwa* embryos; in contrast, their expression appeared undetectable at both stages in SB-treated M*eomesa* embryos (Figure 5C). qRT-PCR results also confirmed that SB treatment significantly inhibited *sox32* and *tbxta* expression in WT and M*hwa* embryos but caused loss of *sox32* and *tbxta* expression in M*eomesa* embryos (Figure 5D). Taken together, these results suggest that Nodal signaling may contribute to mesendoderm induction at variable levels in different genetic backgrounds.

### Maternal *eomesa*, Maternal *hwa* and Nodal Autoregulation Contribute to *ndr1* Expression

**FIGURE 6:**
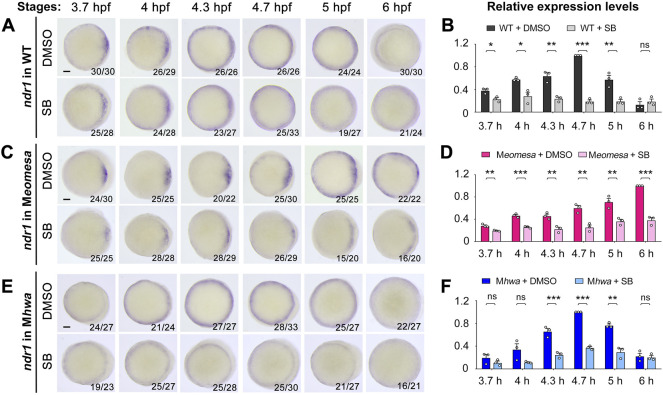
Effect of Nodal signaling inhibition on *ndr1* expression in WT and mutant embryos. WT, M*eomesa* or M*hwa* embryos at the one-cell stage were incubated in Holfreter’s water with 1% DMSO (control) or 50 μM SB431542 (SB) and harvested at indicated stages for detection of *ndr1* expression by WISH **(A,C,E)** or qRT-PCR analysis **(B,D,F)**. Embryos **(A,C,E)** were positioned in animal-pole view with dorsal to the right if the dorsal side was distinguishable. The ratio of embryos with the representative pattern was indicated in the right bottom. Scale bars, 100 μm. qRT-PCR analysis was performed using 15 embryos per sample. The expression level at 3.7, 4, 4.3, 5 and 6 hpf in WT or M*hwa* embryos was normalized to that at 4.7 hpf in WT embryo, while the expression level at different stages in WT or M*eomesa* embryos was normalized to that at 6 hpf in WT embryos. Error bars indicated S.D. based on three biological replicates (indicated by small circles). Statistically significant levels: ns, nonsignificant; *, *p* < 0.05; **, *p* < 0.01; ***, *p* < 0.001.

**FIGURE 7:**
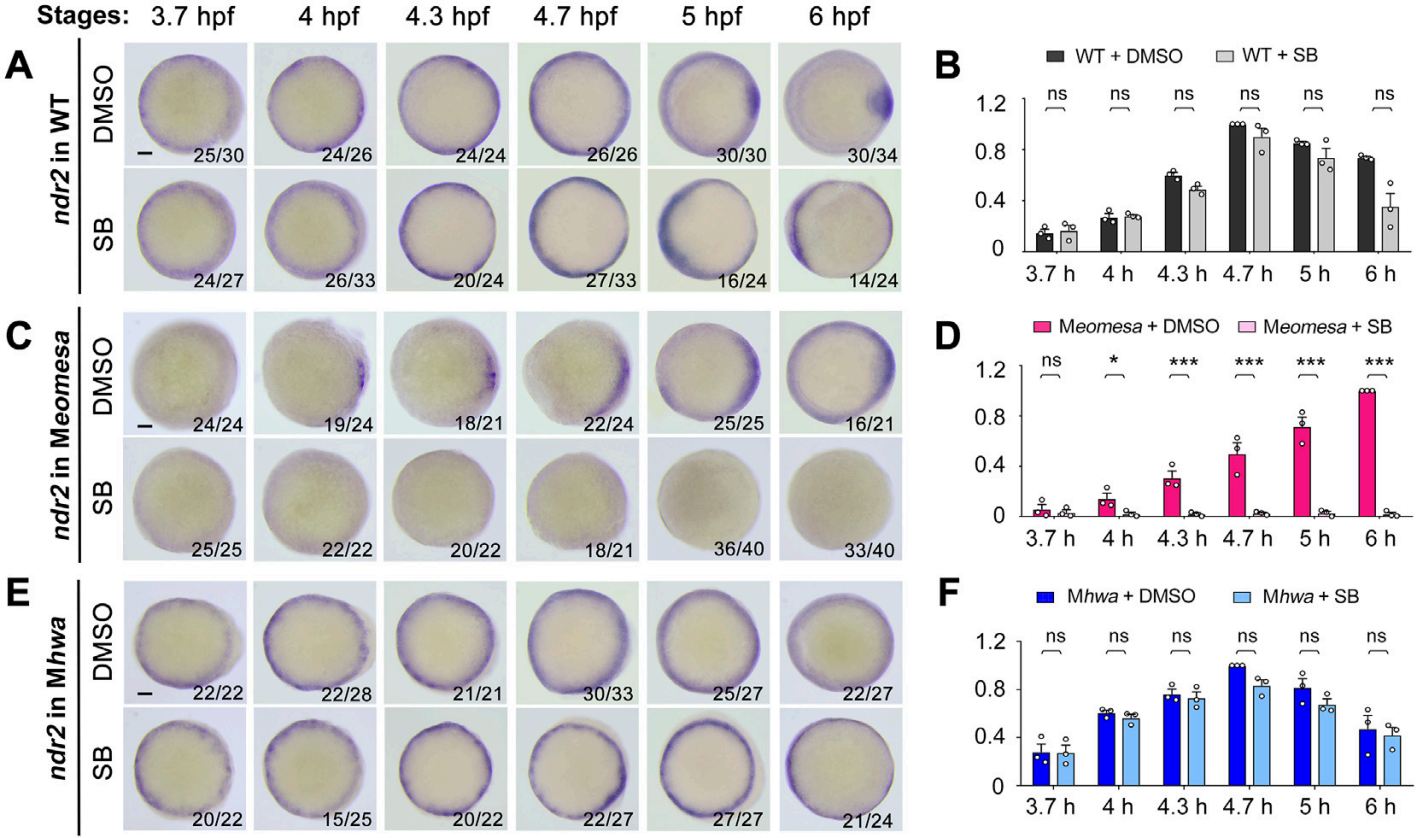
Effect of Nodal signaling inhibition on *ndr2* expression in WT and mutant embryos. Embryo treatment and data presentation were similar to those described in Figure 6 legend.

Based on the above data, we hypothesize that the mesendodermal fate in the zebrafish embryo is induced *via* Ndr1 and Ndr2 by three factors, i.e., maternal *eomesa*, maternal *hwa*-activated β-catenin signaling and Nodal autoregulation. We assume that zygotic *ndr1* and *ndr2* expression in M*eomesa* mutants depends on maternal *hwa* and Nodal autoregulation while their expression in M*hwa* mutants relies on maternal *eomesa* and Nodal autoregulation. To assess contributions of individual factors, we examined *ndr1* and *ndr2* expression patterns by WISH as well as their total levels by qRT-PCR analysis in WT, M*eomesa* and M*hwa* embryos from 3.7 hpf to 6 hpf without or with SB treatment (Figures 6, 7).

We first investigated *ndr1* expression in detail. In SB-treated WT embryos, the expression pattern of *ndr1* was unaltered as indicated by WISH (Figure 6A), but its expression level decreased from 3.7 hpf to 6 hpf as examined by qRT-PCR (Figure 6B), which suggest a role of Nodal autoregulation in maintaining *ndr1* expression. Interestingly, the degree of *ndr1* reduction due to SB treatment gradually increased from 3.7 hpf to 4.7 hpf, implying that Nodal autoregulation contributes to *ndr1* expression more and more during that period.

In M*eomesa* mutants without SB treatment, *ndr1* expression was activated in the dorsal margin with a smaller area than in WT embryos at 3.7 hpf, then propagated ventrally with a slower rate than in WT embryos, and occurred in the whole blastodermal margin at 5 hpf (Figure 6C, upper panel), which were consistent with previous observation (Xu P. et al., 2014). In SB-treated M*eomesa* mutants, in contrast, *ndr1* was still activated in the dorsal margin at a reduced level at 3.7 hpf, its expression domain expanded ventrally but never occupied the whole margin (Figure 6C, lower panel). qRT-PCR results showed that SB-treatment caused a significant reduction (by 31%–62%) of the *ndr1* expression level from 3.7 hpf to 6 hpf (Figure 6D). Thus, in the absence of maternal *eomesa*, maternal *hwa* alone can activate *ndr1* expression in the dorsal margin; and the ventral expansion of *ndr1* expression domain as well as increments of *ndr1* expression heavily depend on positive feedback of Nodal signaling.

In M*hwa* mutants without SB treatment, *ndr1* expression was not prominent in the dorsal margin but similar to WT in the other marginal areas at 3.7 hpf, and its expression pattern later on was comparable to WT embryos (Figure 6E, upper panel; and also see Figure 2C). In SB-treated M*hwa* mutants, *ndr1* expression pattern was not obviously altered at all examined stages as detected by WISH (Figure 6E, lower panel); however, qRT-PCR results showed a significant reduction of *ndr1* expression level at 4.3 hpf, 4.7 hpf and 5 hpf while changes at other stages were not significant (Figure 6F). Apparently, maternal Eomesa alone is capable of activating *ndr1* expression in the whole blastoderm margin but its enhancement during late blastulation requires the contribution of Nodal autoregulation.

Taken together, these results suggest that maternal *eomesa* can activate *ndr1* expression in the whole blastodermal margin, maternal *hwa* activates *ndr1* only in the dorsal margin, and Nodal autoregulation contributes to enhancement of *ndr1* expression.

### *ndr2* Expression Mostly Relies on Maternal *eomesa*

We similarly investigated implication of maternal *eomesa*, maternal *hwa* and Nodal autoregulation in *ndr2* expression. In SB-treated WT embryos, the *ndr2* expression pattern was not obviously altered from 3.7 hpf to 4.7 hpf (Figure 7A). However, unlike in untreated WT embryos, *ndr2* expression in SB-treated WT embryos at 5 hpf and 6 hpf was not prominently enriched in the dorsal margin, instead it appeared enhanced in the ventrolateral margin, for which we did not find an explanation at the moment. The total expression level of *ndr2* in SB-treated embryos, as revealed by qRT-PCR analysis, was not significantly decreased at all examined stages (Figure 7B). It appears that Nodal autoregulation is less important for *ndr2* expression than for *ndr1* expression in WT embryos.

In M*eomesa* mutants without SB treatment (Figure 7C, upper panel), *ndr2* expression occurred in the dorsal margin from 4 hpf to 5 hpf and extended to the whole margin at 6 hpf as reported before (Xu P. et al., 2014). In SB-treated M*eomesa* mutants, however, *ndr2* expression was hardly detectable by WISH (Figure 7C, lower panel). qRT-PCR results showed that, compared to untreated M*eomesa* mutants, the *ndr2* expression level in SB-treated M*eomesa* mutants was almost abolished (Figure 7D). This observation implies that, in the absence of maternal *eomesa*, maternal *hwa* might initiate *ndr1* expression in the dorsal margin at low levels and existing Ndr1 thereof promotes *ndr2* expression through Nodal signaling feedback.

As previously shown in Figure 2D, overall level of *ndr2* expression in M*hwa* was comparable to that in WT embryos. Surprisingly, we observed that the expression pattern and overall level of *ndr2* in M*hwa* embryos was unchanged by SB treatment (Figures 7E,F). Taken together, these data strongly suggest that maternal *eomesa* plays a major role in activation and maintenance of *ndr2* expression in WT background.

## Discussion

**FIGURE 8:**
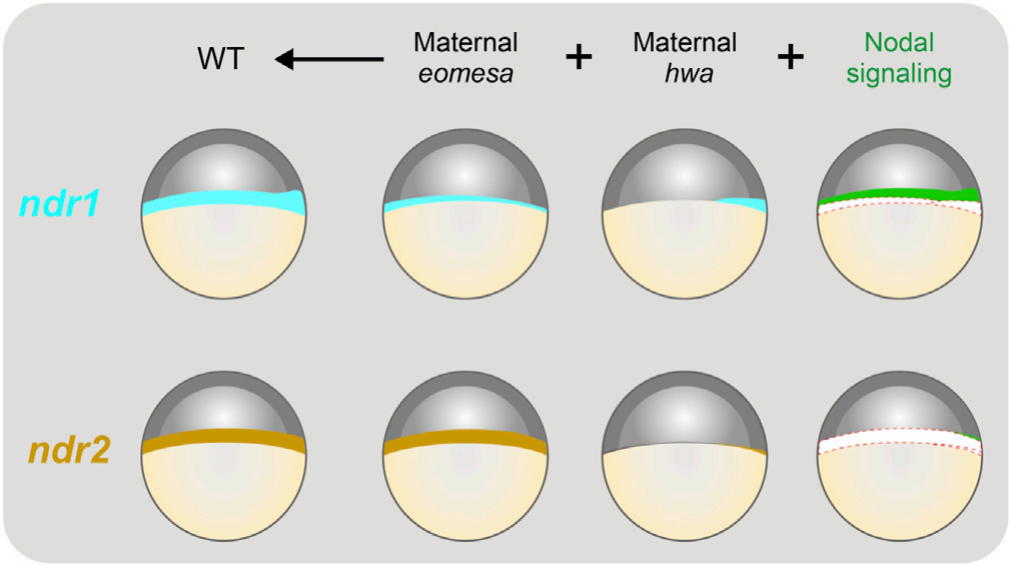
Illustration of contributions of maternal *eomesa*, *hwa*/β-catenin signaling and Nodal signaling to *ndr1* and *ndr2* expression. The expression pattern of *ndr1* and *ndr2* in the late blastula (in lateral view with dorsal to the right and animal pole to the top) is depicted. The overall expression level of *ndr1* or *ndr2* is the sum of contributions from maternal *eomesa* (as seen in M*hwa* without Nodal signaling), maternal *hwa*/β-catenin signaling (as seen in M*eomesa* without Nodal signaling) in and Nodal signaling (autoregulation). In wildtype (WT) embryos, all of the three forces make a significant contribution to *ndr1* expression; however, maternal *eomesa* makes a predominant contribution to *ndr2* expression while maternal *hwa*/β-catenin signaling may contribute a little to *ndr2* expression by activating *ndr1* in the dorsal blastodermal margin and thereof Nodal signaling. In the last column, *ndr1* and *ndr2* levels contributed by *eomesa* and *hwa* were shown as empty to highlight the contribution of Nodal signaling.

In this study, we delineated the roles of maternal *eomesa*, maternal Hwa-activated β-catenin signaling and Nodal autoregulation in spatiotemporal regulation of *ndr1* and *ndr2* expression during early development of zebrafish embryos. As illustrated in Figure 8, maternal *hwa* contributes to *ndr1* expression in the dorsal blastodermal margin, maternal *eomesa* promotes *ndr1* expression throughout the blastodermal margin, and positive feedback of Nodal signaling enhances *ndr1* expression (Figure 8, upper panel). By contrast, *ndr2* expression mostly relies on maternal *eomesa* with minor contribution of maternal *hwa* and Nodal autoregulation during initial activation (Figure 8, lower panel). However, when maternal Eomesa is absent as the case in M*eomesa*, Nodal signaling feedback, which is most likely derived from existing Ndr1, contributes more to *ndr2* expression. Given that *hwa* is maternally expressed only (Yan et al., 2018) and MZ*eomesa* and M*eomesa* mutants show the same *ndr1* and *ndr2* expression patterns before the shield stage (Xu P. et al., 2014), it is unlikely that zygotically expressed *eomesa* and *hwa* transcripts participate in activation of zygotic *ndr1* and *ndr2* expression.

The mouse genome contains a single *Nodal* gene. Its expression may start in the inner cell mass of blastocysts, well before the onset of gastrulation (Granier et al., 2011; Papanayotou et al., 2014), and will be gradually restricted to the posteroproximal region of the primitive streak at the onset of gastrulation (Shen, 2007). Based on transgenic reporter assay, early expression of *Nodal* in mouse blastocysts has been suggested to require the pluripotency factor Oct4, Activin/Nodal signaling and β-catenin signaling (Granier et al., 2011; Papanayotou et al., 2014). A previous study demonstrated that the mouse *Nodal* locus contains an upstream Eomes binding site and overexpression of zebrafish *eomesa* promotes the expression of endogenous *Nodal* gene with mesendoderm induction in murine embryonic stem cells (Xu P. et al., 2014). Given that Eomes protein is expressed in mouse oocytes and early embryos (McConnell et al., 2005), maternal Eomes likely participates in early *Nodal* gene activation in the mouse blastocyst, which needs to be explored in the future.

We observed that the expression of either *ndr1* or *ndr2* in M*eomesa*;M*hwa* double mutant embryos is completely abolished (Figures 2C,D), suggesting that maternal *eomesa* and maternal *hwa* are two essential factors for *nodal* genes expression. Consequently, none of the double mutants showed the expression of the endodermal marker *sox32* (Figures 2A,B), implying that endoderm induction totally depends on Nodal signaling. However, 25–28% of the double mutants retained *tbxta* expression episodically in the blastodermal margin of the double mutants (Figure 2A). It is likely that the mesodermal fate, or *tbxta* expression only, might be induced by other factors or compensatory signaling pathway(s) in the complete absence of Nodal signaling. Our observations are consistent with the fact that *ndr1;ndr2* double mutants completely lack endodermal tissues but still have some posterior mesodermal tissues (Feldman et al., 1998).

A puzzling observation is that inhibition of Nodal signaling has a little effect on *ndr2* expression in WT or M*hwa* embryos (Figures 7A,B,E,F), which suggests a minor or negligible role of Nodal autoregulation in *ndr2* expression. However, in M*eomesa* mutant embryos, Nodal autoregulation makes an obvious contribution to *ndr2* expression (Figures 7C,D). A possible explanation is that association of Eomesa with the general transcription machinery at the *ndr2* locus may mask the Nodal responsive element(s), and these elements can be bound by the Nodal effectors Smad2/3 only when Eomesa is unavailable. The abandonment of Nodal autoregulation for *ndr2* expression might facilitate spatial control of *ndr2* expression domain and function.

Initiation of zygotic *ndr1* and *ndr2* expression occurs after MBT (Rebagliati et al., 1998). However, Eomesa protein exists in the cytoplasm of oocytes and fertilized eggs (Bruce et al., 2003) and *hwa* is also maternally expressed (Yan et al., 2018). An interesting question is why maternal Eomesa and/or maternal Hwa are unable to activate *ndr1* and *ndr2* expression well before MBT. The timing of the zygotic genome activation (ZGA) is proposed to be controlled by the nucleocytoplasmic ratio or the maternal-clock (Tadros and Lipshitz, 2009; Schulz and Harrison, 2019). It remains elusive which ZGA mechanism is adopted by Eomesa/Hwa-activated *ndr1* and *ndr2* expression.

## Data Availability

The original contributions presented in the study are included in the article, further inquiries can be directed to the corresponding author.
